# Decreased Self-Appraisal Accuracy on Cognitive Tests of Executive Functioning Is a Predictor of Decline in Mild Cognitive Impairment

**DOI:** 10.3389/fnagi.2016.00120

**Published:** 2016-07-04

**Authors:** Carole S. Scherling, Sarah E. Wilkins, Jessica Zakrezewski, Joel H. Kramer, Bruce L. Miller, Michael W. Weiner, Howard J. Rosen

**Affiliations:** ^1^School of Psychology, University of San FranciscoSan Francisco, CA, USA; ^2^Memory and Aging Center, Department of Neurology, University of California San FranciscoSan Francisco, CA, USA; ^3^School of Nursing, Duke UniversityDurham, NC, USA; ^4^Department of Radiology, School of Medicine, University of California San FranciscoSan Francisco, CA, USA

**Keywords:** dementia, anosognosia, Alzheimer’s disease, neuropsychology, prodromal symptoms, disease progression, neurodegeneration, cognition

## Abstract

**Objective**: Mild cognitive impairment (MCI) in older individuals is associated with increased risk of progression to dementia. The factors predicting progression are not yet well established, yet cognitive performance, particularly for memory, is known to be important. Anosognosia, meaning lack of awareness of one’s impaired function, is commonly reported in dementia and is often also a feature of MCI, but its association with risk of progression is not well understood. In particular, self-appraisal measures provide an autonomous measure of insight abilities, without the need of an informant.

**Methods**: The present study examined the utility of self-appraisal accuracy at baseline for predicting cognitive decline in 51 patients using an informant-free assessment method. Baseline task performance scores were compared to self-assessments of performance to yield a discrimination score (DS) for tasks tapping into memory and executive functions.

**Results**: Linear regression revealed that a larger DS for executive function tasks in MCI predicted functional decline, independent of age, education, and baseline memory and executive task scores.

**Conclusion**: These findings indicate that objective estimates of self-appraisal can be used to quantify anosognosia and increase predictive accuracy for decline in MCI.

## Introduction

Cognitive decline is a common feature of aging (Anstey and Low, 2004; Weaver et al., 2006). In many individuals cognitive changes represent the early signs of neurodegenerative disease that will ultimately progress to dementia. Much of the research aimed at improving early diagnosis of neurodegenerative disease has focused on the syndrome of mild cognitive impairment (MCI). MCI is defined as a syndrome of cognitive change in the elderly that sometimes represents a transitional phase between normal aging and dementia (Petersen et al., 1999; Celsis, 2000; Collie and Maruff, 2000). Studies of patients with MCI have revealed that, among the many potential markers predicting decline, performance on neuropsychological testing is one of the most powerful (Tabert et al., 2006; Albert et al., 2007; Dickerson et al., 2007; Howieson et al., 2008; Saxton et al., 2009; Summers and Sauders, 2012). Most of these studies have highlighted the importance of episodic memory tasks, but several groups have indicated that tasks tapping other domains of cognition can improve predictive accuracy (Baudic et al., 2006; Tabert et al., 2006; Dickerson et al., 2007; Howieson et al., 2008; Libon et al., 2010). One domain that has received relatively little study is metacognition, or awareness of one’s own cognitive abilities and impairments (Eslinger et al., 2005; Spitznagel and Tremont, 2005).

Anosognosia, or awareness of one’s deficits, is a common feature of dementia which becomes more prevalent and severe as the illness worsens (Mullen et al., 1996; Agnew and Morris, 1998; Zanetti et al., 1999; Vogel et al., 2005; Rosen et al., 2010; Maki et al., 2012). Because many patients with MCI complain about their poor memory, anosognosia is not considered a central feature of MCI (Edmonds et al., 2014). A number of studies, however, have demonstrated that MCI is associated with anosognosia by using tools to compare patients’ assessments of their own abilities with appraisals of knowledgeable informants. Such methods have demonstrated that some MCI patients overestimate their cognitive abilities (Albert et al., 1999; Tabert et al., 2002; Vogel et al., 2004, 2005). Furthermore, studies using these informant based methods have demonstrated that anosognosia in MCI is associated with an increased risk of decline on longitudinal follow-up (Tabert et al., 2002; Edmonds et al., 2014). These findings are supported by other studies indicating that informant reports are more predictive of cognitive decline in MCI compared with self-report (Tierney et al., 1996; Gifford et al., 2014). Taken together, these data indicate that measurement of anosognosia in MCI is useful for predicting decline to dementia.

Methods for quantifying anosognosia vary. While most studies compare self-report to informant reports, it is possible to compare a person’s appraisal of their abilities to objective measurements based on neuropsychological testing (Clare, 2004; Clare et al., 2004; Williamson et al., 2010; Rosen, 2011). These methods have potential advantages over informant-based measures due to additional factors which might influence informant estimates, including the informant’s familiarity with the patient as well as their own cognitive function and emotional states. Additionally, knowledgeable and reliable informants may not be available in many clinical and research settings. At least two studies have demonstrated anosognosia in MCI without the use of informants (Rosen et al., 2010; Krueger et al., 2011); however, it has yet to be demonstrated whether such impairment predicts decline to dementia. The goal of this analysis was to evaluate the utility of neuropsychologically-based measurements of self-appraisal to predict cognitive decline in MCI on longitudinal follow-up. Because memory is a known predictor of cognitive decline in MCI, we wished to establish whether self-appraisal of impairments had value beyond what would be predicted by baseline memory scores. We thus included two memory assessments in our analysis: one to predict decline based on memory-task performance and the second to predict decline based on memory self-appraisal. In addition, because deficits in MCI may include multiple domains of cognition, especially in executive functioning processes such as attention, planning and judgment (Traykov et al., 2007; Brandt et al., 2009; Gomar et al., 2011; Johns et al., 2012), we evaluated self-appraisal for executive performance.

## Materials and Methods

### Participants

Subjects were recruited from a larger study of MCI, the goal of which was to identify risk factors for decline. The self-appraisal tasks were added part-way through the recruitment phase, and were administered to all enrollees from this point when time permitted. The study began recruitment in 2003 and participants in the study were followed for an average of about 3 years. Participants were referred to the study by memory clinics in the San Francisco Bay Area, including the Memory Disorders Clinic at the San Francisco Veterans Affairs Medical Center, the Memory and Aging Center (MAC) at the University of California, San Francisco, and the Memory Disorders Clinic at the California Pacific Medical Center or recruited by flyers and advertisements in local newspapers. Participants were required to have an informant who knew them well and could answer questions about their cognition and general health. Exclusion criteria included any medical, psychiatric, or neurologic condition (other than MCI) that could significantly affect brain structure or cognition. All data included in this manuscript was obtained in compliance with UCSF CHR system and written informed consent was in accordance with the Declaration of Helsinki and UCSF CHR system.

The larger study enrolled both cognitively normal participants and those with MCI. MCI was operationally defined MCI according to published guidelines (Petersen, 2004; Winblad et al., 2004). To capture the broadest range of MCI, we did not require that subjects perform below specific cutoffs on psychometric testing because we were interested in including individuals at the mildest end of the MCI spectrum (i.e., those with Clinical Dementia Rating (CDR) sum of boxes scores 0.5–1). If cognitive testing did not reveal significant impairment (>1.5 standard deviations below age-matched norms), both the participant and informant had to endorse subjective cognitive decline. Healthy controls (HC) were defined as those having a CDR of 0. Since we aimed to assess the relationship between self-appraisal at baseline and subsequent decline, we included individuals who had a diagnosis of MCI at baseline as well as those diagnosed as cognitively HC (non-controls, NC) at baseline who subsequently declined to the point of MCI or dementia on longitudinal follow-up. We were able to identify 51 individuals (ALLsample) meeting these criteria in whom the self-appraisal task had been administered, including nine NC and 42 MCI at baseline. At final assessment, seven NC received a new diagnosis of MCI, two NC were diagnosed with dementia (1 Alzheimer’s disease, AD; 1 behavioral variant frontotemporal dementia, bvFTD), 22 MCI maintained a diagnosis of MCI, and 20 MCI progressed to AD. Overtime, 29 participants underwent a change in clinical diagnosis and 22 maintained an MCI diagnosis.

### Neuropsychological and Functional Assessment

All participants completed a battery of standardized assessments including: the Mini-Mental State Examination (MMSE, Folstein et al., 1975), modified Trails (Number, Letter, Switching between numbers and letter; Kramer et al., 2003), Stroop (Color naming, Word naming, Inhibition, Switch; Delis et al., 2001), Wechsler Memory Scale, immediate and verbal delayed recall (WMS; Wechsler, 1997), and the California Verbal Learning Test-2nd Edition (CVLT-2; Delis et al., 2000) long delayed free recall (Ldfr) and recognition (Rgn). The CDR Morris, 1993) was used to quantify functional impairment.

### Self-Appraisal

**Figure 1:**
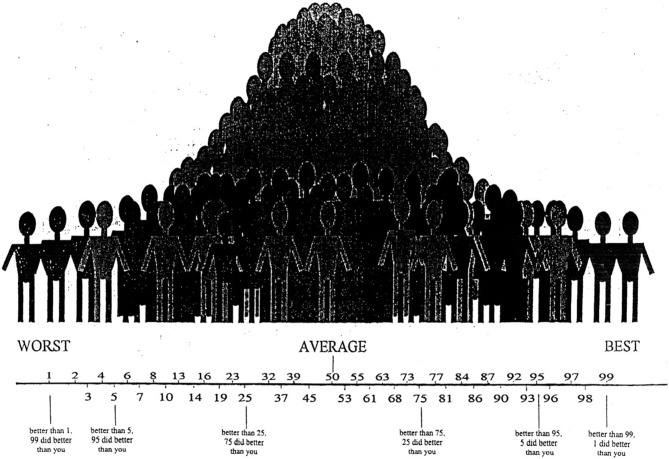
**Picture of the bell curve provided as a visual aid for the self-appraisal component of assessment, with labels corresponding to percentile rankings ranging from 1 to 99.** Prior to the memory and executive tasks, participants were informed that they were going to do a post-task performance assessment, rating themselves on the task compared to what they would expect to be average performance. In addition, they were consistently reminded that on most tasks the majority of people would score in the average range, at 50th percentile, while fewer individuals would have much higher or much lower scores than average.

Self-appraisal accuracy was evaluated by comparing self-ratings to actual performance for the modified Trails switch condition, Stroop switch condition and WMS long delay tasks (see “Analysis” Section for creation of accuracy scores). The CVLT-2 was not used for self-appraisal as it was used as an independent memory performance score to predict decline. At the beginning of the testing session, patients were informed that they would be asked to rate their performance compared to what they would expect to be average performance. They were reminded that on most tasks the majority of people would score in the average range, at 50th percentile, while fewer individuals would have much higher or much lower scores than average. A picture of a bell curve was provided as a visual aid, with labels corresponding to percentile rankings ranging from 1 to 99 (Figure 1). This method has been used to assess self-appraisal in adults with neurodegenerative disease (Rosen et al., 2010; Williamson et al., 2010) and cognitive impairment due to HIV (Chiao et al., 2013) as well as in children with cognitive impairment (Krueger et al., 2011). Self-assessment was only requested after completion of each task, because other studies have shown that prediction of performance on typical neuropsychological tasks is poor even in normal adults (Eslinger et al., 2005).

### Analysis (Figure 2)

Self-appraisal was measured for both executive function and episodic memory at the baseline testing session. For each of these tasks, a discrimination score (DS) was computed by subtracting actual performance (Pr) from self-appraised performance (SAPr): DS = SAPr-Pr. The WMS long delay recall score was used to create the memory DS score (memDS). A composite DS for executive function (execDS) was created using the mean DS from the switch subtests of the Trails and Stroop tasks. Because the DS is calculated based on actual performance, it is important to control for this performance to accurately assess the effect of self-appraisal. In theory, if all subjects rated themselves as exactly average, then the variability in DS would only represent variability in performance and not self-appraisal. To control for memory performance (memPr), we used the CVLT-2 Ldfr score. To control for executive function performance, we used an executive function composite score (execPr) created by averaging the scores from the remaining five executive subtests (Stroop color, number and inhibition; Trails number and letter).

**Figure 2:**
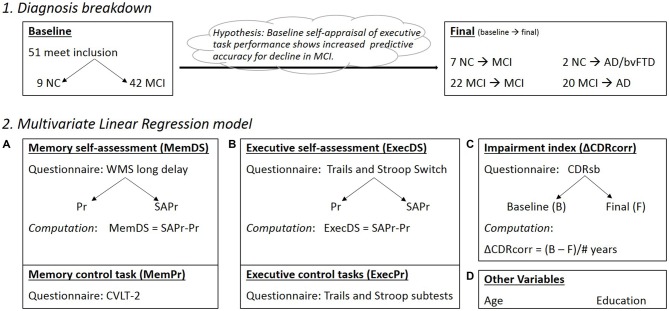
**(1)** Diagnosis breakdown for the ALLsample at Baseline and Final testing sessions (Note: Final box indicates conversions from baseline status); NC, normal controls; MIC, mild cognitive impairment; AD, Alzheimer’s disease; bvFTD, behavioral variant frontotemporal dementia. **(2)** Components making up the regression model (Pr, performance; SAPr, Self assessment of performance; DS, Discrimination score). **(A)** Memory component of the model, with DS and Pr control scores; **(B)** Executive component of the model, with DS and Pr control scores; **(C)** Impairment index corrected for the variable times between baseline and final diagnosis; **(D)** Other important variables in the model, including Age at baseline assessment and Education level (# of years).

The CDR “sum of boxes” (CDRsb), measured at each testing session, provided an index of functional impairment, where a higher number represents larger burden. The outcome variable of interest was functional change over time as measured by change in the CDRsb score. This was used instead of likelihood of conversion to dementia in order to capture progressive decline in function not severe enough to merit a diagnosis of dementia. Change in CDRsb (ΔCDRsb) was calculated for each patient by subtracting the score at baseline from the score at the last assessment. Because the duration of follow-up varied across individuals, a correction factor for time was applied (ΔCDRcorr= ΔCDR/number of years between assessments) to create an annualized rate of decline for CDRsb.

Statistical analyses were computed using SPSS (version 22.0; SPSS/IBM, Chicago, IL, USA). Normality for individual variables was determined by the Shapiro–Wilk test. Distributions were normal for all variables except for the ΔCDRcorr. A natural log transform was applied to normalize this data. A multivariate linear regression examined the relationship between the potential predictors, including memDS, memPr, execDS, execPr scores, and the outcome ΔCDRcorr, with age and education included as additional covariates. Added variance contributed by the main factors of interest, execDS and memPr scores, were examined through separate stepwise multivariate linear regression analyses.

## Results

Baseline descriptive statistics, ΔCDRcorr and neuropsychological testing data are presented in Table 1. As would be expected based on prior characterization of MCI, performance in this group was worse for memory (41st percentile) than for executive function (55th percentile). On average, patients tended to self-rate their memory performance as poor, as indicated by a slightly negative memDS (−2.16 percentile points), but over-rated their executive function performance at 15.7 percentile points better than their actual performance.

**Table 1:** **Mean values for the “ALL” cohort (*n* = 51)**.

	Age	Education	Sex	Test Years	MMSE	ΔCDRcorr	execPr	execDS	memPr	memDS
**Mean**	75.80	17.02	35M/16F	3.00	28.39	0.81	55.24	15.72	41.31	−2.16
**Standard deviation**	7.16	2.66	—	1.87	1.66	0.87	22.95	32.19	36.13	32.62
**Min/Max**	55/89	8/22	—	0.72/7.45	0.07/1.49	0.50/6.50	2.60/92.60	−46.5/81.50	0.00/99.00	−75/88

*Age = age of participant at baseline; Education = years; Test Years = years between the baseline and final testing session; MMSE, Mini Mental State Examination; ΔCDR, Change in CDR sb (final − baseline); ΔCDRcorr = annual rate of decline of ΔCDR; execPr = Actual task performance on executive tasks (Stroop: color, word, inhibition; Trails: number, letter); execDS = discrimination score for executive tasks (Stroop and Trails: switch subtests); memPr = actual task performance (CVLT-2 delayed recall); memDS = memory discrimination score (WMS verbal delayed recall)*.

Linear regression using six predictors (execDS, execPr, memDS, memPr, age and education) in the ALLsample (*n* = 51) accounted for 25.5% of the variance in functional decline, *F*_(6,44)_ = 3.847, *p* = 0.004, R^2^ = 0.344, R^2^ adjusted = 0.255, 90% CI [−7.482, −1.944]. Both memPr and execDS scores were independent predictors of decline. MemPr (or CVLT-2 scores) was inversely related to decline (lower score, more decline), while execDS was positively correlated with decline, indicating that those who rated themselves best relative to their actual performance showed more decline (Table 2A). Stepwise regressions indicate that memPr scores explain 11% and ExecDS contributes to 6.7% of the total model variance, calculated using the Adjusted R^2^ values.

**Table 2:** **Multiple regression analysis on log transformed ΔCDRcorr**.

	Zero-order correlations (R)	Standard coefficients beta (R^2^)	*t*	Significant	90% CI lower (F)	90% CI upper Significant
			Adjusted R^2^			
**A. “ALL” cohort (*n* = 51)**
(Constant)			-2.860	0.006	-7.482	-1.944
Exec_Pr	−0.258	0.107	0.581	0.564	−0.008	0.017
Exec_DS	0.336	0.415	2.245	0.030	0.003	0.021
Memory_Pr	−0.375	−0.372	−2.758	0.008	−0.016	−0.004
Memory_DS	0.124	−0.123	−0.888	0.380	−0.010	0.003
Age	0.343	0.259	2.008	0.051	0.006	0.063
Education	0.080	0.239	1.849	0.071	0.008	0.162

Total model	0.587	0.344	0.255		3.874	0.004

**B. “MCIonly” cohort (*n* = 42)**
(Constant)			−2.436	0.020	−9.304	−0.845
Exec_Pr	−0.2240	0.221	0.958	0.345	−0.010	0.028
Exec_DS	0.301	0.510	2.219	0.033	0.001	0.029
Memory_Pr	−0.348	−0.408	−2.511	0.017	−0.022	−0.002
Memory_DS	0.149	−0.164	−0.968	0.340	−0.015	0.005
Age	0.291	0.263	1.1729	0.093	0.007	0.084
Education	0.087	0.192	1.314	0.197	−0.038	0.179

Total model	0.558	0.311	0.193		2.636	0.032

Age = age of participant at baseline; Education = years; ΔCDRcorr = annual rate of decline of ΔCDR; execPr = Actual task performance on executive tasks (Stroop: color, word, inhibition; Trails: number, letter); execDS = discrimination score for executive tasks (Stroop and Trails: switch subtests); memPr = actual task performance (CVLT delayed recall); memDS = memory discrimination score (WMS verbal delayed recall). *significant at p < 0.05.

Furthermore, we considered the possibility that including individuals who started as NC but declined over time could detract from the clinical relevance of the analysis. Such patients would not be identified in a clinic as having a potential neurodegenerative disease, and thus the question of what predicts decline in them would not come up in practice. Thus, we repeated the regression analysis with only those who began the study with an MCI diagnosis (MCIonly, *n* = 42). The results were similar, with the overall model accounting for 19.3% of the variance in functional decline, *F*_(6,35)_ = 2.636, *p* = 0.032, R2 = 0.311, R adjusted = 0.193, 90% CI [−9.304, −0.845]. Again, both memPr and execDS were independent predictors of decline (Table 2B). Stepwise regressions in the MCIonly cohort uncovered individual variance in the larger model for memPr and ExecDS scores, explaining 11.9% and 8.8%, respectively.

## Discussion

The present study used an objective neuropsychologically-based assessment battery along with an informant-free, subjective self-appraisal task to demonstrate that reduced insight into performance abilities at baseline predicts a larger degree of functional decline in MCI. The findings were specific to executive function and were independent of other variables of interest including age, education, executive task performance, memory task performance and self-appraisal of memory abilities. The results suggest that objective estimates of self-appraisal in patients themselves, using defined ranking measurements, could be used to quantify anosognosia and increase predictive accuracy for decline in MCI.

Our finding that baseline self-appraisal impairment predicts a higher likelihood of decline in MCI is consistent with prior studies demonstrating that patients with MCI tend to overestimate their skills (Tierney et al., 1996; Albert et al., 1999; Vogel et al., 2004; Orfei et al., 2010), and that the discrepancy between the ratings of the patient and those of informants is predictive of decline to AD. A recent study of MCI in the Alzheimer’s Neuroimaging Initiative came to a similar conclusion when they found that the informant estimate of a patient’s function is more valuable than the patient’s estimate in predicting decline. Underestimating one’s function has also been found to be associated with cerebrospinal fluids (CSF) markers indicating AD (Edmonds et al., 2014). The majority of studies examining anosognosia in MCI, including all those looking at predictors of decline, have used informant or caregiver accounts of patient abilities. The approach taken in the current analysis has potential advantages in that it can be used when an informant is not available and does not depend on the informant to “notice” sometimes subtle changes in the patient. Additionally, a task-based protocol with immediate self-assessment questions permits cognitive-domain specificity, limiting overgeneralization of abilities when self-appraisals are only collected at the end of a lengthy testing session. A possible disadvantage of this technique is potential loss of validity in more cognitively impaired patients who may have difficulty understanding or remembering the instructions to rate oneself in terms of percentile. This is less of a concern in MCI patients, whose cognitive dysfunction is mild compared with dementia patients. In theory, the concept of percentile rankings may be impacted by education, but it should be noted that we considered education in the multiple regression analysis and we have previously used this method in children as young as 12 and identified relationships with teacher/parent behavioral ratings (Krueger et al., 2011). Further studies in adults with varying levels of education would be helpful in establishing the generalizability of this approach.

Memory impairments are considered to be one of the first reported deficits in MCI and AD patients, yet executive changes are known to be affected in progressed dementia and considered to be among the earliest cognitive changes in AD (Wilson et al., 1983; Storandt and Hill, 1989; Albert, 1996; Dickerson et al., 2007). Decreased performance on similar executive tasks have been shown to be predictors of progressively diminishing functional abilities over time (older vs. young adults: Spieler et al., 1996; healthy elderly vs. mild AD: Castel et al., 2007; APOE-4 status MCI vs. non-APOE MCI: Albert, 1996). Such executive tasks require large frontal contributions for successful completion (Trails: Ettlin et al., 2000; Stuss et al., 2001; Stroop: Peterson et al., 2002; Ridderinkhof, 2002; De Pisapia and Braver, 2006) a region also reported as showing atrophy or hypometabolism in patients with dementia (Rahman et al., 1999; Snowden et al., 2002). Similarly, previous work has indicated that insight impairments are also a likely a function declining frontal lobe integrity (Mendez and Shapira, 2005; Mimura and Yano, 2006; Salmon et al., 2006; Rosen et al., 2010; Williamson et al., 2010).

Our study found that self-appraisal for executive function, but not memory function, predicted decline. This makes sense considering that the most common complaint in MCI patients is memory impairment. This was evident in our cohort, where initial memory performance was low (41st percentile) and the discrepancy between actual performance and self-appraisal was only 2 percentile points. In contrast, executive function was in the average range in the group as a whole, but the discrepancy was much larger (15 percentile points). This indicates that the patients were sensitive to their memory decline, but overestimated their executive function capabilities (some to a very high degree). This raises the possibility that the brain’s capacity for self-monitoring is not equal for all domains of cognition. Memory monitoring may be intrinsically stronger than monitoring of executive function, which may partly explain why memory complaints are among the most common in aging as well as MCI (Busse et al., 2006; Albert et al., 2007; Dickerson et al., 2007). Indeed, prior studies have suggested that self-monitoring varies across domains of neurological function (McGlynn and Schacter, 1989).

Some notable limitations warrant discussion. The small sample size of “converted” patients, who received a clinical diagnosis of dementia over time, restricted power to compute our multifactorial comprehensive multiple-regression analysis on this specific cohort. However, with a conversion rate of 16% in our longitudinal sample, this is comparable to general MCI to AD conversion rates (10–15%; Caroline, 2008; Mridula et al., 2008). In addition, race and sex biases are also potential limitations, with a 94% Caucasian population and a 2–1 male dominated cohort. The latter sex difference warrants further study in MCI and dementia cohorts, since previous studies in healthy samples have shown that men tend to inflate self-judgments of task ability compared to women, even when performing on the same level (Mengelkamp and Jäger, 2007). Finally, the voluntary recruitment nature of our sample and the affiliation with a “memory” clinic lead to a pre-selection bias in this patient cohort. These patients are more aware of personal cognitive impairments, leading to seek clinical help and participate in such studies. The contributing values of insight deficits presented in this article are likely underestimated and worse in the general affected population.

Overall, while previous research has suggested measures of memory as a sensitive measure of MCI deficits and likelihood of progression, self-appraisal deficits related to executive capacities may be more sensitive, particularly early in disease progression. This latter point is important in prodromal forms of dementia, such as MCI. Intrinsic variation in self-monitoring abilities across domains may make executive function a more sensitive target for detecting self-appraisal impairments indicating more severe disease or greater likelihood of cognitive decline. In addition, it is possible that domain-specificity in self-appraisal has other important implications; for instance, it may be a better marker of functional impairment. The ability to assess domain-specific self-appraisal in a well-defined manner is another potential advantage for neuropsychologically-based approaches compared with informant based approaches for measuring anosognosia. For this reason, as well as others discussed above, we believe that these approaches should be used more frequently in formal cognitive assessments of patient cohorts with possible progressive disease burden, such as MCI.

## Author Contributions

Each author contributed to the current manuscript at different parts of project set-up and data acquisition. CSS and HJR ran the statistics and wrote the manuscript. Each author has revised and approved the current version of this article. All authors listed, have made substantial, direct and intellectual contribution to the work, and approved it for publication.

## Conflict of Interest Statement

Unless otherwise noted below, the authors have no conflict of interest to report. J. Kramer has authorship for the article proposing the CVLT, 2nd edition assessment which was used in this study. Dr. Weiner has served on the Scientific Advisory Boards for Pfizer, BOLT International, Neurotrope Bioscience, Eli Lilly, U. of Penn’s Neuroscience of Behavior Initiative, National Brain Research Centre (NBRC), India, LEARN Program at University of North Carolina, Dolby Family Ventures, LP, and ADNI. He has provided consulting to Synarc, Pfizer, Janssen, KLJ Associates, Easton Associates, University of California, Los Angeles (UCLA), Alzheimer’s Drug Discovery Foundation (ADDF), Neurotrope Bioscience, Avid Radiopharmaceuticals, Clearview Healthcare Partners, Perceptive Informatics, Smartfish AS, Decision Resources, Inc., Araclon, Merck, Defined Health, Howard University, Biogen Idec, BioClinica, Genentech, and Howard University. The following entities have provided funding for travel; Pfizer, Neuroscience School of Advanced Studies (NSAS), Kenes, Intl., ADRC, UCLA, UCSD, Sanofi-Aventis Groupe, University Center Hospital, Toulouse, Araclon, AC Immune, Nutricia, Eli Lilly, New York Academy of Sciences (NYAS), National Brain Research Center, India for Johns Hopkins Medicine, Consortium for Multiple Sclerosis Centers (CMSC), Northwestern University, Fidelity Biosciences Research Initiative, University of Pennsylvania, The Alzheimer’s Association, Merck, ADPD, and Biogen Idec. He served on the Editorial Boards for Alzheimer’s & Dementia and MRI. Dr. Miller receives grant support from the NIH/NIA and the Centers for Medicare & Medicaid Services (CMS) as grants for the Memory and Aging Center. As an additional disclosure, Dr. Miller serves as Medical Director for the John Douglas French Foundation; Scientific Director for the Tau Consortium; Director/Medical Advisory Board of the Larry L. Hillblom Foundation; and Scientific Advisory Board Member for the National Institute for Health Research Cambridge Biomedical Research Centre and its subunit, the Biomedical Research Unit in Dementia (UK).
